# Monitoring algorithm of hospitalized patients in a medical center with SARS-CoV-2 (Omicron variant) infection: clinical epidemiological surveillance and immunological assessment

**DOI:** 10.7717/peerj.14666

**Published:** 2023-01-23

**Authors:** Chi-Sheng Chen, Ming-Jr Jian, Chih-Kai Chang, Hsing-Yi Chung, Shih-Yi Li, Jung-Chung Lin, Kuo-Ming Yeh, Ya-Sung Yang, Chien-Wen Chen, Shan-Shan Hsieh, Sheng-Hui Tang, Cherng-Lih Perng, Feng-Yee Chang, Hung-Sheng Shang

**Affiliations:** 1 Division of Clinical Pathology, Department of Pathology, Tri-Service General Hospital, Taipei, Taiwan; 2 Division of Infectious Diseases and Tropical Medicine, Department of Medicine, Tri-Service General Hospital, Taipei, Taiwan; 3 Division of Pulmonary and Critical Care Medicine, Department of Medicine, Tri-Service General Hospital, Taipei, Taiwan

**Keywords:** B.1.1.529, BA.2, Anti-N IgM, Anti-N IgG, VOC genotyping, Epidemiological surveillance, SARS-CoV-2, Immunology, COVID-19 immunoglobulin

## Abstract

**Purpose:**

Coronavirus disease 2019 (COVID-19), caused by severe acute respiratory syndrome coronavirus 2 (SARS-CoV-2), is a major healthcare threat worldwide. Since it was first identified in November 2021, the Omicron (B.1.1.529) variant of SARS-CoV-2 has evolved into several lineages, including BA.1, BA.2–BA.4, and BA.5. SARS-CoV-2 variants might increase transmissibility, pathogenicity, and resistance to vaccine-induced immunity. Thus, the epidemiological surveillance of circulating lineages using variant phenotyping is essential. The aim of the current study was to characterize the clinical outcome of Omicron BA.2 infections among hospitalized COVID-19 patients and to perform an immunological assessment of such cases against SARS-CoV-2.

**Patients and Methods:**

We evaluated the analytical and clinical performance of the BioIC SARS-CoV-2 immunoglobulin (Ig)M/IgG detection kit, which was used for detecting antibodies against SARS-CoV-2 in 257 patients infected with the Omicron variant.

**Results:**

Poor prognosis was noted in 38 patients, including eight deaths in patients characterized by comorbidities predisposing them to severe COVID-19. The variant-of-concern (VOC) typing and serological analysis identified time-dependent epidemic trends of BA.2 variants emerging in the outbreak of the fourth wave in Taiwan. Of the 257 specimens analyzed, 108 (42%) and 24 (9.3%) were positive for anti-N IgM and IgG respectively.

**Conclusion:**

The VOC typing of these samples allowed for the identification of epidemic trends by time intervals, including the B.1.1.529 variant replacing the B.1.617.2 variant. Moreover, antibody testing might serve as a complementary method for COVID-19 diagnosis. The combination of serological testing results with the reverse transcription-polymerase chain reaction cycle threshold value has potential value in disease prognosis, thereby aiding in epidemic investigations conducted by clinicians or the healthcare department.

## Introduction

Since late 2019, cases of coronavirus disease 2019 (COVID-19) caused by a novel coronavirus named severe acute respiratory syndrome coronavirus 2 (SARS-CoV-2) have been reported worldwide (Bedford et al., 2020; Wu & McGoogan, 2020). By June 2022, more than 530 million COVID-19 cases were reported globally, which were associated with over six million deaths (https://covid19.who.int/). Mutations arise naturally as a result of viral replication, causing the wild-type strain of SARS-CoV-2 to evolve into several emerging variants of concern (VOC) such as the Alpha (B.1.1.7), Beta (B.1.351), Gamma (P.1), and Delta (B.1.617.2) variants (Tiecco et al., 2022). A new SARS-CoV-2 variant, B.1.1.529, was identified on November 9, 2021, which was named Omicron by the World Health Organization (WHO) on November 26, 2021 (Shy et al., 2022). The SARS-CoV-2 Omicron variant (B.1.1.529) is further divided into several sub-lineages, including BA.1, BA.2, BA.2 sub-lineage, and BA.3 (Mohapatra et al., 2022b; Viana et al., 2022), along with the newly emerging sub-variants BA.4 and BA.5 (Mohapatra et al., 2022c). These VOCs may enhance virus infectivity/transmissibility, reduce vaccine susceptibility and/or disease severity, and cause diagnostic failure (Boehm et al., 2021; Chekol Abebe et al., 2022; Davies et al., 2021; Iketani et al., 2022; Zou et al., 2022). Immediately following the declaration of the Omicron variant as a VOC by the WHO, the Taiwan Central Epidemic Command Center strengthened border control. Subsequently, the first case of the Omicron variant transmitted from South Africa was identified on December 11, 2021. Consequently, Taiwan experienced a surge of cases in its fourth wave of the COVID-19 pandemic in May 2022, which included imported cases and infections spreading among local communities. The SARS-CoV-2 Omicron variant has now rapidly replaced the Delta variant as the dominant lineage in Taiwan. Along with Taiwan, the Omicron variant caused a surge of the COVID-19 pandemic worldwide, including in adjacent countries such as Japan, South Korea, Hong Kong, and Vietnam.

Nucleic acid amplification testing (NAAT) has been the preferred initial diagnostic test for COVID-19 (Kevadiya et al., 2021; Perng et al., 2020). In general, the reverse transcription-polymerase chain reaction (RT-PCR) cycle threshold (*Ct*) value is inversely proportional to the viral load and correlates strongly with the cultivability of the virus (Mishra et al., 2022; Singanayagam et al., 2020). Nevertheless, low viral RNA levels might be interpreted either as a late or early stage of infection. Antibody testing for COVID-19 can serve as a complementary diagnostic tool to RT-PCR. As seroconversion is generally observed 3–14 days after symptom onset (Jiang et al., 2022; Yamamoto et al., 2022) and antibody detection is simpler and faster than viral RNA load testing (Kevadiya et al., 2021), it serves as a promising alternative tool for COVID-19 diagnosis (Feng, 2020). Before vaccination, the detection of any of the nucleocapsid (N) protein, spike (S) protein, or receptor binding domain (RBD) antibodies during SARS-CoV-2 antibody testing indicated previous exposure to SARS-CoV-2. However, owing to the commercialization of vaccines, the history of vaccination and/or prior SARS-CoV-2 infection must be considered when interpreting antibody test results. This has raised an important issue regarding the appropriate use of serologic assays.

Here, we aimed to evaluate the clinical performance of the BioIC SARS-CoV-2 immunoglobin (Ig)M/IgG detection kit for detecting IgM and IgG antibodies against SARS-CoV-2, particularly with respect to the VOC Omicron (B.1.1.529) lineage, which is driving the fourth wave of the Taiwan COVID-19 epidemic. This study highlights the practical application of combining serological testing results with the RT-PCR *Ct* value for disease prognosis, which may help clinical staff or the healthcare department conduct epidemic investigations. With the increasing number of SARS-CoV-2 cases associated with the Omicron sub-lineage, we also aimed to characterize and compare the clinical epidemiology of Omicron BA.2 breakthrough infections among hospitalized COVID-19 cases.

## Materials & Methods

### Study design and clinical specimens

This study was registered on April 4, 2022, and was approved by the Tri-Service General Hospital Institutional Review Board (approval number: C202005041). Paired throat swab and blood samples were collected from each patient with a COVID-19 diagnosis admitted to the hospital between December 2021 and May 2022. The severity of COVID-19 was defined according to the WHO severity classification (World Health Organization (WHO), 2020). Breakthrough infection is considered to occur in an individual who has been fully vaccinated. Given the shifting definitions of what constitutes a “fully vaccinated” individual with the introduction of booster vaccines (Edwards & Orenstein, 2022), in this study, we defined breakthrough infection as a COVID-19 case occurring in an individual 14 or more days after completing the primary series with recommended doses of an authorized vaccine (Centers for Disease Control and Prevention (CDC), 2021; Centers for Disease Control and Prevention (CDC), 2022a). Patients were excluded from our study when their infected virus variants could not be detected or the remained serum samples were insufficient for the serology tests. Informed consent was obtained from all the participants involved in the study.

### RT-PCR testing for SARS-CoV-2 detection

NAAT for SARS-CoV-2 was performed as described previously (Chang et al., 2022; Jian Jr et al., 2021). Briefly, SARS-CoV-2 RT-PCR testing was performed using the LabTurbo AIO 48 system (LabTurbo, New Taipei City, Taiwan) for detecting the SARS-CoV-2 *N1* and *E* genes. A *Ct* value <35 was considered as a positive result for the pathogen. Each sample had an internal control (Rnase *P* gene). The external controls comprised RNA spike-in mix as the positive control and diethylpyrocarbonate-treated water as the negative control.

### SARS-CoV-2 IgM/IgG detection

**Figure 1:**
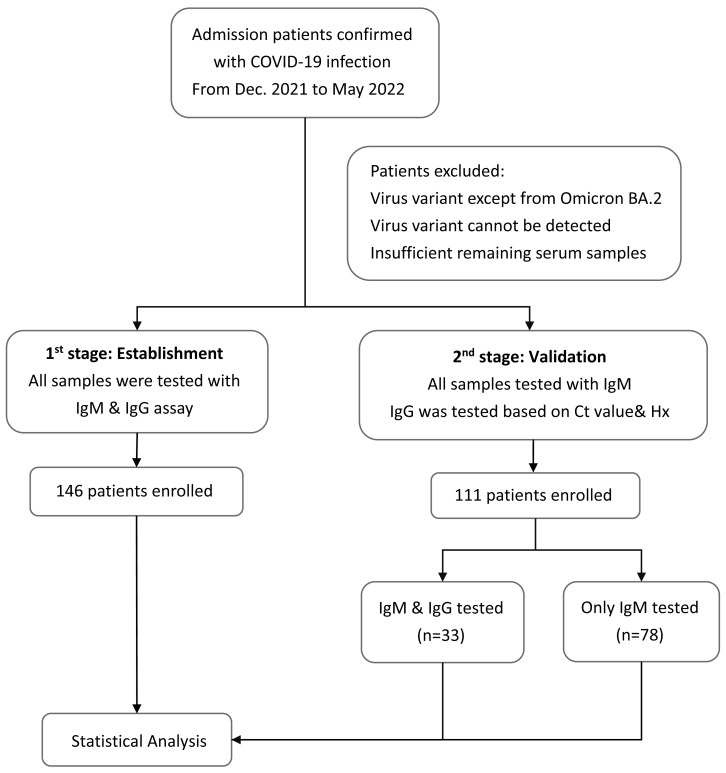
Flowchart of the study design. Flowchart with the number of patients included in each stage of the study to provide more clarity into the methodology and recruitment process.

Plasma samples were collected at the same time when the patients were admitted to the hospital with confirmed cases of COVID-19. IgM and IgG antibodies against SARS-CoV-2 were detected using the BioIC SARS-CoV-2 IgM/IgG detection kit, which has received Emergency Use Authorization by the Taiwan Food and Drug Administration. The BioIC System includes BioIC cartridges and an analyzer. The BioIC cartridge comprises the following three advanced technologies: microfluidic liquid handling, chemiluminescence-based detection, and a multiplexed antibody assay. These technologies work together to simultaneously detect anti-IgG or anti-IgM antibodies against the SARS-CoV-2 spike glycoprotein S1 subunit, S2 subunit, RBD, and N protein. Each BioIC cartridge is pre-equipped with a sample injection aperture, reagent injection aperture, reservoir, and microfluidic network. The protocol was executed according to the manufacturer’s instructions. Test results greater than or equal to the signal-to-cutoff ratio of 1.0 were interpreted as positive for SARS-CoV-2 antibodies; results below the cutoff were considered negative. The patients would be divided into two groups according to the time when they were recruited in our study (Fig. 1). The first stage was to establish the model and helping us find out the relationship between the *Ct* value and serology results during the infection disease course. Therefore, the samples would be tested with both IgM and IgG. Moving to the second stage, rest of the patients would be used to validate our theory and only IgM would be tested for all patients because most patients were in acute infection stage when admission. IgG would only be tested in patients whose disease course cannot be easily classified based on their *Ct* value and medical history.

### SARS-CoV-2 VOC molecular typing

All positive SARS-CoV-2 specimens were screened using six multiplex RT-PCR assays, as described previously (Chung et al., 2022). Briefly, a 20 µL reaction mixture (5 µL RNA and 15 µL PCR master mix) containing the primer/probe mixture was designed to detect nine mutations (ΔHV 69/70, K417T, K417N, L452R, E484K, E484Q, N501Y, P681H, and P681R) against the RBD of the S protein of SARS-CoV-2 variants.

### Statistical analyses

Independent *t*-test was used to compare the normally distributed continuous variable between two groups and a one-sided *P* ≤ .05 was considered significant. Statistical analyses were performed using Excel (Microsoft Corp, Redmond, WA, USA) and GraphPad Prism Version 8.0 (GraphPad, Inc., San Diego, CA, USA).

## Results

### Demographic and clinical information of patients with Omicron infection

During the study period (from December 2021 to May 2022), data were collected for 257 COVID-19 patients at Tri-Service General Hospital, a medical center-level teaching hospital in northern Taiwan. The clinical data of the patients are summarized in Table 1. The median age was 42 years (range: 3–85 years). The *Ct* values for the SARS-CoV-2–positive RT-PCR results ranged from nine to 35. Among the 257 patients with confirmed COVID-19, 62 (24%) were unvaccinated patients while 195 (76%) had received at least one vaccine dose and 96 (37.4%) had received a third booster dose of the vaccine. All SARS-CoV-2 infections were identified to belong to the BA.2 sub-lineage by our novel VOC PCR typing method. More patients were symptomatic (80.2%) than asymptomatic (19.8%). Fifty-one adult or elderly patients showed moderate to severe symptoms, with a median age of 58 years and 70 years, respectively. Most patients were discharged, although eight patients died, all of whom but one had comorbidities predisposing them to more severe COVID-19. Five of these patients were aged above 70 years with comorbidities of type 2 diabetes mellitus, cardiovascular diseases, and chronic respiratory diseases, including chronic obstructive pulmonary disease, bronchial asthma, or chronic hypercapnia. The remaining two patients were immunocompromised due to underlying disease, with one patient having undergone heart transplantation and the other having hypopharyngeal squamous cell carcinoma with lung metastasis.

**Table 1:** Patient characteristics.

	**All patients (*n* = 257)**	**Asymptomatic (*n* = 51)**	**Mild case (*n* = 155)**	**Moderate (*n* = 20)**	**Severe (*n* = 31)**
Age, median (SD), *y*	42 ± 23.42	38 ± 18.62	38 ± 21.61	58 ± 29.17	70 ± 28.53
<11, no, (%)	13 (5.1%)	0	7 (4.5%)	2 (10.0%)	4 (12.9%)
11–18, no, (%)	11 (4.3%)	2 (3.9%)	7 (4.5%)	1 (5.0%)	1 (3.2%)
19–30, no, (%)	62 (24.1%)	18 (35.3%)	42 (27.1%)	1 (5.0%)	1 (3.2%)
31–60, no, (%)	93 (36.2%)	20 (39.2%)	63 (40.6%)	6 (30.0%)	4 (12.9%)
61–70, no, (%)	31 (12.1%)	7 (13.7%)	15 (9.7%)	1 (5.0%)	8 (25.8%)
70–80, no, (%)	20 (7.8%)	1 (2.0%)	12 (7.7%)	2 (10.0%)	5 (16.1%)
>81, no, (%)	27 (10.5%)	3 (5.9%)	9 (5.8%)	7 (35.0%)	8 (25.8%)
Sex					
Male, no, (%)	128 (49.8%)	32 (62.7%)	65 (41.9%)	13 (65.0%)	18 (58.1%)
Female, no, (%)	129 (50.2%)	19 (37.3%)	90 (58.1%)	7 (35.0%)	13 (41.9%)
Medical Hx					
w/o^a^ any medical Hx, no, (%)	139 (54.1%)	31 (60.8%)	98 (63.2%)	6 (30.0%)	4 (12.9%)
T2DM, no, (%)	42 (16.3%)	12 (23.5%)	12 (7.7%)	4 (20.0%)	14 (45.2%)
Cardiovascular disease, no, (%)	132 (51.0%)	34 (66.7%)	70 (45.2%)	9 (45.0%)	19 (61.3%)
Pregnancy, no, (%)	4 (1.6%)	2 (3.9%)	2 (1.3%)	0	0
Malignancy, no, (%)	15 (5.8%)	4 (7.8%)	4 (2.6%)	4 (20.0%)	3 (9.7%)
Autoimmune disease, no, (%)	28 (10.9%)	13 (25.5%)	13 (8.4%)	1 (5.0%)	1 (3.2%)
Chronic virus infection, no, (%)	30 (11.7%)	8 (15.7%)	9 (5.8%)	5 (25.0%)	8 (25.8%)
Vaccination					
None vaccinated, no, (%)	62 (24.1%)	3 (5.9%)	18 (11.5%)	16 (6.2%)	25 (80.6%)
1 dose, no, (%)	11 (4.3%)	3 (5.9%)	7 (4.5%)	1 (5.0%)	0
2 dose, no, (%)	88 (34.2%)	24 (47.1%)	64 (41.3%)	0	0
3 dose, no, (%)	96 (37.4%)	21 (41.2%)	66 (42.6%)	3 (15.0%)	6 (19.4%)
Ct value, median (SD)	17 ± 5.89	24 ± 6.77	16 ± 5.07	14 ± 3.10	18 ± 6.24

**Notes.**

a w/o, without.

### Anti-N IgM and anti-N IgG serological testing

Anti-N IgG and IgM were measured for all 146 patients divided into first stage to establish our model, using the BioIC IgM/IgG detection kit. The resting patients divided to second stage were all tested with Anti-N IgM and only 33 patients were tested with the anti-N IgG.

**Figure 2:**
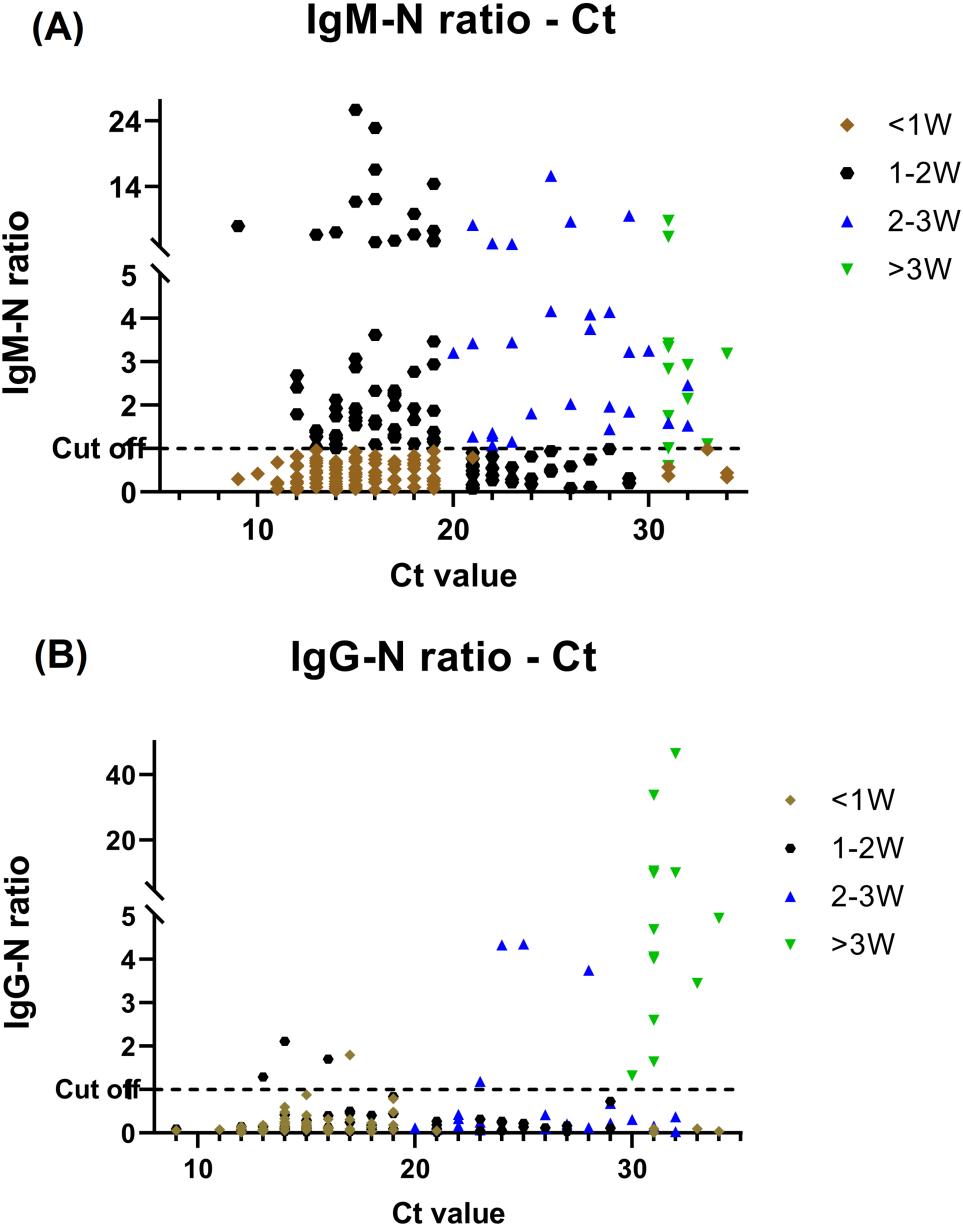
BioIC antibody screening results *versus* cycle threshold (*Ct*) value from patients with COVID-19. (A) BioIC anti-N Immunoglobulin (Ig)M *versus Ct* value. (B) BioIC anti-N IgG *versus Ct* value. BioIC anti-N IgG/IgM signal-to-cutoff ratio of 1.0. <1W, symptom onset <1 week; 1–2W, symptom onset between 1 and 2 weeks; 2–3W: symptom onset between 2 and 3 weeks; >3W, symptom onset >3 weeks; *Ct* value, cycle threshold value.

Of the 257 specimens, 108 (42%) and 24 (9.3%) were positive for anti-N IgM and IgG respectively. We classified patients to different period during their disease courses based on the serology findings and *Ct* value and comparative investigation of the serological testing and RT-PCR assay results is shown in Fig. 2. Prominent Anti-N IgM seroconversion was found in the majority of patients after one week of the disease course. Furthermore, when comparing the data of each group of the disease course, the elevation of the IgM titer between <1 week and other groups had statistics significance (*P* ≤ .05) by using independent *t*-test. The anti-N IgG seroconversion was evident >2–3 weeks after infected but the statistics significance (*P* ≤ .05) was only found in those who be infected more than 3 weeks when comparing to others.

### Relationship between *Ct* value and infection disease course

**Figure 3:**
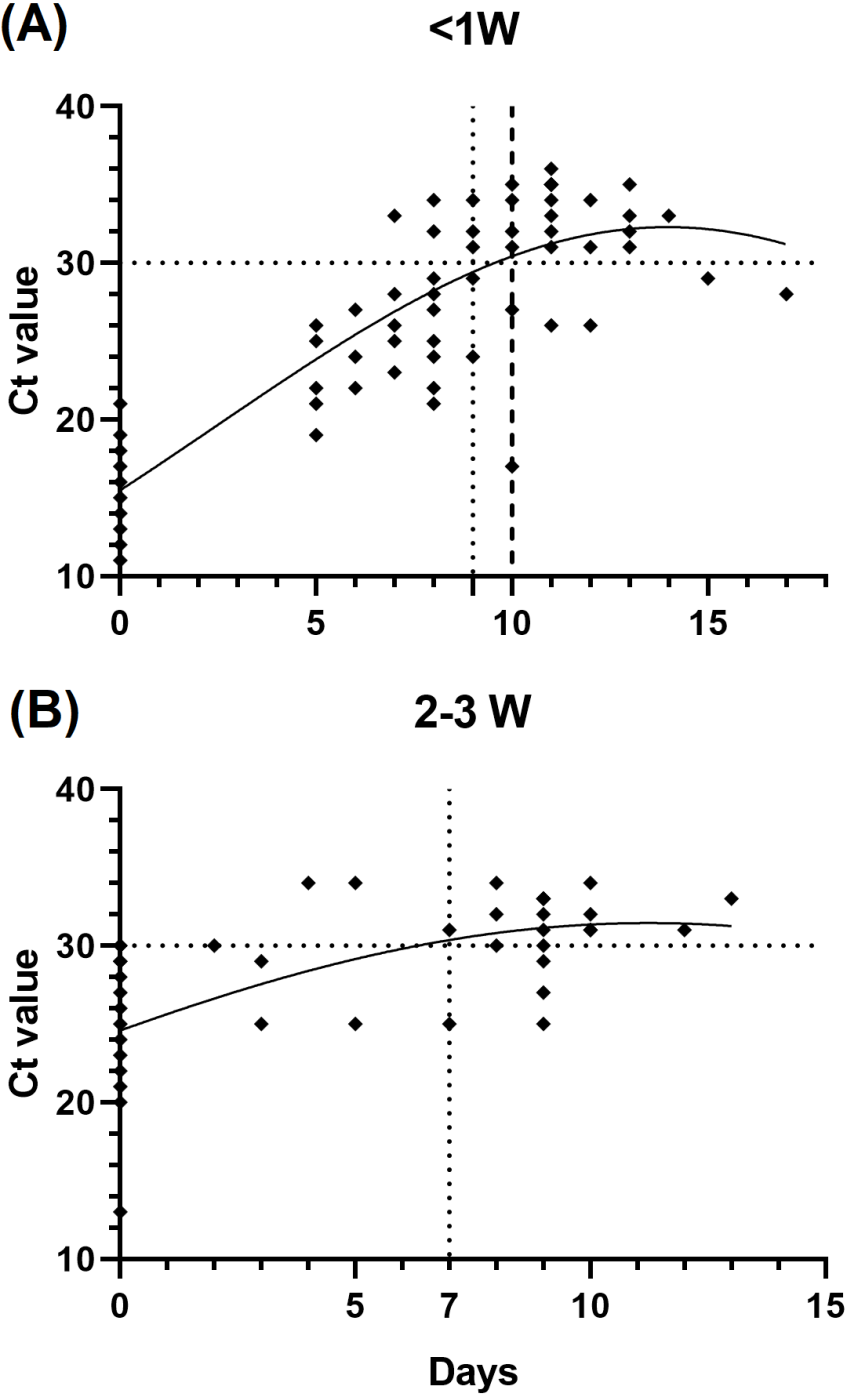
Relationship between cycle threshold (*Ct*) value and symptom onset interval. (A) <1 week since symptom onset patients *versus Ct* value. (B) 2–3 weeks since symptom onset patients *versus Ct* value.

To infer the relationship of the immunological assessment and the infection disease course, we evaluated the changes of the patients’ *Ct* value during hospitalization, which is correlated with the duration of infectiousness. We found that patients who be infected less than 1 week prior to testing (*n* = 29) showed slower viral load decline than those after 2 to 3 weeks (*n* = 17), as we defined a *Ct* value >30 as the standard for release from hospital isolation (Fig. 3).

### Combined antibody and RT-PCR testing in the prognosis of COVID-19

**Figure 4:**
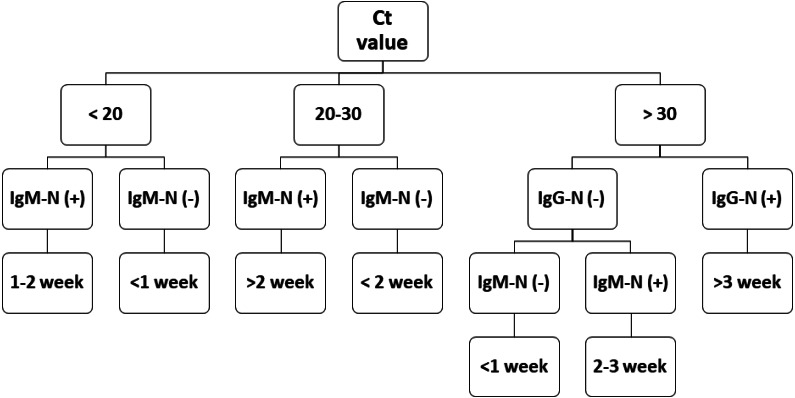
COVID-19 prognostic evaluation flow chart.

During epidemic investigations, it is important to categorize the reported COVID-19 cases as recent/acute infection or late infection. The *Ct* value has an inverse relationship with viral load. Although a low *Ct* value indicates acute infection, a higher *Ct* value is less applicable. Antibody tests can serve as a vital complementary method for epidemic investigations. The flowchart in Fig. 4 demonstrates that combining the results of RT-PCR and antibody testing might help clinicians better evaluate the disease course more quickly and apply corresponding medical measures precisely. This approach may also aid health departments in improved formulation of quarantine standards and epidemic investigations.

## Discussion

Our study provides insight into the disease course of COVID-19 regarding the antibody kinetics for hospitalized patients. We found that the antibody kinetics of anti-N IgG/IgM tests in COVID-19 patients offer valuable information in predicting the disease course. A lower *Ct* value at 1 week after infected could indicate a longer hospital stay (Figs. 2 and 3). SARS-CoV-2 VOCs have emerged as a global threat. Molecular diagnostic tools, including whole-genome sequencing and PCR-based typing, provide effective information in improving infection control management and epidemiological surveillance during the COVID-19 pandemic. Taiwan encountered the fourth infection wave due to emergence of the Omicron variant in December 2021, while infections due to other variants decreased dramatically. In our study, we also observed that infections due to the BA.2 variant increased considerably after February 2022, which might become the dominant variant in Taiwan’s future epidemic. This study also highlights the importance of VOC typing, which had a great impact on implementing public health strategies to counter these mutation strains.

Individuals in most developed countries, including Taiwan, have received two complete doses of the COVID-19 vaccine or even additional booster doses (Mohapatra et al., 2022a; Ritchie et al., 2020). Immunization provides neutralizing antibodies against SARS-CoV-2, especially during the emergence of VOCs such as the Omicron variant (Burki, 2022; Collie et al., 2022; Garcia-Beltran et al., 2022; Muik et al., 2022). However, vaccine breakthrough infections have still been reported in fully vaccinated individuals (Dimeglio et al., 2022; Juthani et al., 2021; Vignier et al., 2021; Zhang et al., 2022). In our study, most patients had received at least one vaccine dose, with some receiving the third booster dose; however, they were still infected with the Omicron variant. Vaccinated individuals might harbor neutralizing antibodies against SARS-CoV-2, which could decrease disease severity and reduce transmissibility or hospitalization rate/duration, as previously reported (Brosh-Nissimov et al., 2021; Tenforde et al., 2021). The symptoms caused by Omicron infections are reported to be clinically milder, whereas those with comorbidities or unvaccinated individuals may have higher risk of developing complications or severe COVID-19 (Kahn et al., 2022; Mahase, 2021). Similarly, in our study, patients with comorbidities or those who were unvaccinated had a poor outcome. The most severe cases of COVID-19 occurred in elderly patients with underlying conditions (80%) in our study. Previous reports showed that an impaired early inflammatory response was associated with older age (Xie et al., 2021), which might explain why these patients had a significantly elevated viral load and delayed humoral response compared to younger patients. Unvaccinated patients, including those who had received only one dose of a two-dose primary series, developed more severe symptoms than those who were vaccinated with the full primary series. Other reports also demonstrated that unvaccinated individuals were at a higher risk of becoming infected and of COVID-19-associated death (Johnson et al., 2022; Machado et al., 2022). This study thus further highlights the necessity of full vaccination and recommended booster doses to best protect against the Omicron variant pandemic.

The humoral response against SARS-CoV-2 mainly targets the S and N viral proteins (Chamkhi et al., 2022). Here, we screened for anti-N IgM as an epidemic investigation of patients with COVID-19 based on local infection or imported cases. The supplementary anti-IgM test has been reported to provide better sensitivity in the diagnosis of COVID-19 (Guo et al., 2020; Higgins et al., 2021) prior to the availability of vaccines. However, the appropriate use of serologic testing requires further elucidation (Centers for Disease Control and Prevention (CDC), 2022b). With the increase in vaccination coverage, some serologic antibody testing against anti-S1, S2, or RBD of the spike protein (S1) mighty not be suitable to diagnose a COVID-19 infection status. The classification of the disease course may consist of bias due to lacking information in medical records and it was more difficult for asymptomatic patients to determine when being infected for symptom onset was not available., On the other hand, it could demonstrate the value of the anti-N IgG/IgM level in helping evaluating the COVID-19 disease course.

There are also several limitations of this study that should be considered when interpreting the results. First, complete information might not be available or necessarily reliable, and the temporal data are only depicted as days since an RT-PCR–positive diagnosis of COVID-19. Second, the included cohort was limited by the sample size of the patients who were admitted at our hospital, which may not be representative of the general population. Finally, there are many variants of SARS-CoV-2 circulating, and we only focused on Omicron as the VOC that emerged during this study period; further studies are thus warranted to determine whether other variants exhibit the same dynamics found in this investigation.

## Conclusions

This study provides valuable information regarding the detection of anti-N IgG/IgM antibodies in patients with COVID-19 for inference of the clinical disease course. The choice to study IgG and IgM against the SARS-CoV-2 N protein was made for practical reasons, since vaccination status would not interfere with detection of this antibody. That is, since most vaccines target the S protein, evaluating IgG and IgM against SARS-CoV-2 S protein would influence the judgment of disease progression. The combination of serum antibody and RT-PCR results not only increased the positive rate to enable an early diagnosis of COVID-19 infection but also helped us rapidly predict the disease course of the patients. Such early diagnosis is one of the most effective methods to control the spread of the virus, enabling early treatment and early isolation of patients.

Overall, SARS-CoV-2 VOCs have emerged as a global threat. Implementing a PCR-based method to quickly detect known VOCs is necessary for molecular epidemiological surveillance. Furthermore, by combining the approach of serological anti-N IgG and IgM testing along with nucleic acid tests, clinical workers can more easily decipher the disease course and act appropriately. This combined testing approach can also serve as a powerful tool for epidemic investigations and as a guide to improve public health measures.

##  Supplemental Information

10.7717/peerj.14666/supp-1 Data S1 Raw data

## Additional Information and Declarations

### Data Availability

The following information was supplied regarding data availability: The raw data is available in the Supplemental File.
